# The Effects of a Reproductive Health Voucher Program on Out-of-Pocket Family Planning and Safe Motherhood Service Expenses: A Yemeni Study

**DOI:** 10.3390/healthcare13131591

**Published:** 2025-07-03

**Authors:** Omar Z. Al-Sakkaf, El-Morsy A. El-Morsy, Shaimaa A. Senosy, Al Shaimaa Ibrahim Rabie, Ahmed E. Altyar, Rania M. Sarhan, Marian S. Boshra, Doaa M. Khalil

**Affiliations:** 1 Department of Public Health and Community Medicine, Faculty of Medicine and Health Sciences, University of Aden, Aden City, Yemen; omeralzain@yahoo.com; 2 Department of Public Health and Community Medicine, Faculty of Medicine, Beni-Suef University, Beni-Suef 62521, Egypt; elmorsy50@yahoo.com (E.-M.A.E.-M.); shoshoahmed80@yahoo.com (S.A.S.); 3 Clinical Pharmacy Department, Faculty of Pharmacy, Beni-Suef University, Beni-Suef 62521, Egypt; alshaimaa.ph@o6u.edu.eg (A.S.I.R.); raniamohammad87@yahoo.com (R.M.S.); mariansobhy31@yahoo.com (M.S.B.); 4 Department of Pharmacy Practice, Faculty of Pharmacy, King Abdulaziz University, P.O. Box 80260, Jeddah 21589, Saudi Arabia; 5 Pharmacy Program, Batterjee Medical College, P.O. Box 6231, Jeddah 21442, Saudi Arabia

**Keywords:** safe motherhood and family planning voucher program, reproductive health, Yemen

## Abstract

**Background/Objectives:** Using healthcare vouchers mitigates the financial burdens of low-income individuals, therefore enhancing mothers’ satisfaction and encouraging service utilization. In Yemen, reducing financial barriers results in marked improvement in reproductive health services utilization for mothers and their newborns. Such financial strain can be addressed through reproductive health vouchers, which reduce out-of-pocket expenses of family planning, pregnancy, birth, postnatal care and neonatal care. This study compares the Safe Motherhood and Family Planning Voucher Program in the Lahj governorate to the non-voucher program in the Abyan governorate in terms of enhancement of reproductive healthcare accessibility and use. **Methods**: This facility-based, quantitative, comparative, cross-sectional study was conducted in the Lahj governorate, which supports the Safe Motherhood and Family Planning Voucher Program, and the Abyan governorate, which does not. **Results**: The voucher-supported program has greatly improved mothers’ satisfaction, access, and use of all reproductive health services by covering transportation, covering lodging during hospitalization, and providing free reproductive treatments. Compared to Abyan mothers, Lahj governorate mothers more frequently used rental vehicles (paid for by the voucher program) and free reproductive health services (*p*-value < 0.001). Lahj governorate mothers (who used the vouchers) used family planning, prenatal care, facility-based delivery, home delivery by competent birth staff, cesarean section, postnatal care, and neonatal care more frequently than Abyan governorate mothers. A health institution which supported the Safe Motherhood and Family Planning Voucher Program (SMHFPVP) provided prenatal care (98.5%), competent birth services (99.0%), and modern contraceptive use (92.3%)—oral contraceptive pills, implants, injectables, contraceptive patches, vaginal rings, and intrauterine devices—for mothers who were interviewed and attended the targeted HFs in the Lahj governorate, compared with (77.6%), (80.3%), and (67.8%), respectively, for mothers in Abyan governorate who were not supported by the SMHFPVP. This study demonstrates substantially higher satisfaction levels among voucher-using mothers in the Lahj governorate compared to those in the Abyan governorate, across all satisfaction domains and overall satisfaction scores. **Conclusions**: This study found that women without access to maternal health vouchers experienced worse prenatal, natal, and postnatal care and were less satisfied with healthcare services compared with women who used vouchers.

## 1. Introduction

Unfortunately, fragile and conflict-affected nations fell behind in terms of attaining the Millennium Development Goals (MDGs). The Sustainable Development Goals (SDGs) do not consider the distinctive hurdles these nations face, especially when it comes to providing their citizens with essential services [1,2]. Weak and failing institutions, extreme poverty and inequality, and the global expansion of violence and conflict are among the numerous factors that contribute to national vulnerability. Along with the dynamic nature of healthcare needs, the unpredictability of healthcare expenses, and regional variations in morbidity and mortality rates, humanitarian crises pose unique challenges that call for creative responses [3].

The supply of healthcare and the facilitation of access to services present substantial obstacles for governments in these conditions, since pregnant mothers and their newborns face unique health risks in states ravaged by violence [4]. Yemeni women experience various forms of violence, particularly during the displacement crisis, in which over 3 million Yemeni citizens have become internally displaced persons (IDPs). This situation has significantly impacted maternal and infant health, leading to increased morbidity and mortality rates among mothers and their infants. Atrocities, forced migration, and other natural disasters account for almost 60% of maternal deaths, 53% of child deaths, and 45% of infant deaths worldwide [5].

Women and their families can receive healthcare more easily with the support of vouchers for safe motherhood programs, which also channel critical funds to service providers, allowing them to improve the quality of their services. Healthcare vouchers have a long history of providing underserved populations with access to valuable services [6,7].

Yemen has been on the list of fragile nations and conflict-affected regions since 2010, and it is one of the poorest countries in the Middle East and North Africa (MENA) region [4]. Notably, public health facilities in rural areas of Yemen are underfunded, whereas most private healthcare facilities are located in urban areas, particularly within the main cities of governorates and their districts, resulting in relatively better accessibility to health services in urban areas. These facilities often cater to individuals with higher disposable incomes.

There are myriad financial hurdles that affect underprivileged mothers, such as the cost of maternal health treatments, as well as travel and subsistence expenses while they are away from home, all of which incur significant costs. The life and health of pregnant women and their fetuses are put at great risk when they travel to a distant health facility, considering factors such as high and unpredictable expenses, lengthy distances, and unsafe traveling conditions [4]. A significant number of women do not have access to family planning, resulting in a heightened risk of unplanned pregnancies in environments that may endanger their health and well-being. Consequently, an estimated 75,000 pregnant Yemeni women are in danger of experiencing difficulties due to the critical condition of health services in the nation [8].

The Reproductive Health Voucher Program in Yemen, funded by the German government through the Kreditanstalt für Wiederaufbau (KfW), has been running since 2013 and serves two governorates: Ibb in the central region and Lahj in the southern region. As a result of the conflict, the program remained suspended from 2015 until the end of 2018. At the present time, serious food insecurity is afflicting Lahj, one of the poorest governorates in southern Yemen [4]. Nevertheless, the governorate has benefited from a relatively functional public health system and committed health staff engaged in discovering novel ways to improve the health of Lahj residents [9]. This study targeted two governorates: the Lahj governorate, which is supported by the SMHFPVP, and the Abyan governorate, which is not.

The Lahj governorate is characterized by the following features:

Geography and Climate:

Lahj is situated in southwestern Yemen, bordered by the Red Sea to the west and the Arabian Sea to the south. The governorate encompasses diverse landscapes, including coastal plains, fertile valleys, and mountainous regions. The climate varies from arid in the lowlands to temperate in the highlands.

Demographics and Society:

The population of Lahj is predominantly Arab, with a rich cultural heritage influenced by various historical periods. The society is organized into tribal communities, each with its own customs and traditions. The urban centers are characterized by a mix of modern and traditional architecture, while rural areas maintain a more traditional lifestyle.

Economy and Livelihood:

Agriculture is the backbone of Lahj’s economy, with the fertile Wadi Tuban valley supporting the cultivation of crops such as sorghum, sesame, and vegetables. Livestock farming is also prevalent, with sheep and goats being the most common. Fishing along the coastline provides an additional source of income for coastal communities.

History and Politics:

Historically, Lahj was part of the Sultanate of Lahj, a protectorate under British colonial rule until 1967. The region has experienced various political changes, and its strategic location has made it a focal point in Yemen’s modern history.

The Abyan governorate is characterized by the following features:

Geography and Climate:

Abyan is located in southern Yemen, bordering the Arabian Sea. The governorate features a mix of coastal plains, fertile valleys, and mountainous terrain. The climate is generally hot and arid, with seasonal variations influencing agricultural practices.

Demographics and Society:

The Abyan population is primarily Arab, with a strong presence of tribal communities. The social structure is deeply rooted in tribal affiliations, which play a significant role in governance and community life. The region has a history of political instability, affecting its social dynamics.

Economy and Livelihood:

Agriculture is a key economic activity in Abyan, with the fertile valleys supporting the cultivation of crops like bananas, dates, and vegetables. Livestock farming and fishing are also important, contributing to the livelihood of many residents. However, the economy has faced challenges due to conflicts and environmental factors.

History and Politics:

Abyan has a complex history, with periods of conflict and political upheaval. It was once part of the Fadhli Sultanate. The governorate has experienced significant political changes, influencing its development and stability.

Table 1 shows the sociodemographic and health characteristics of mothers of reproductive age (15–49) in the Lahj and Abyan governorates.

N.B.: Despite the sociodemographic similarities between Abyan and Lahj, as demonstrated in Table 1, any bias introduced by differences in socioeconomic characteristics between the two governorates would tend to favor Abyan, since Abyan residents fall within a higher wealth quintile compared to Lahj residents, and the literacy rate is much higher in Abyan (which has a slightly higher economic status and literacy percentage).

Local voucher distributors find eligible women in the community, educate them about safe motherhood services, and offer vouchers for Yemeni Rial (YER) 200 (less than USD 1) [10]. About 15% of vouchers are totally subsidized for individuals who cannot pay this low charge. The voucher provides free access to four antenatal care (ANC) appointments; safe delivery, including in the case of pregnancy-related complications and cesarean section; two postnatal care (PNC) visits; two neonatal care (NNC) visits and postnatal family planning. The package includes free transportation to a hospital, as well as lodging for companions and food, if necessary.

In the current study, we assessed the effectiveness of the Safe Motherhood and Family Planning Voucher Program in improving the access and utilization of reproductive health services in the Lahj governorate through examining the uptake of specific RH services among women in the health facilities studied, compared to in the Abyan governorate, where the program is not supported.

## 2. Materials and Methods

### 2.1. Study Type, Location, and Period

This quantitative, facility-based, analytical, cross-sectional study was designed to investigate the effectiveness of the SMHFPVP in improving access to and utilization of reproductive health services (RHSs) in the Lahj governorate, where the program was active and supporting health facilities, compared to in the Abyan governorate, which was not supported by the SMHFPVP. The Lahj governorate was chosen since it was the only governorate in the southeastern region that conducted the program, while the Abyan governorate, another southeastern governorate, was not supported by the SMHFPVP and is similar to the Lahj governorate in terms of sociodemographic characteristics, the types and distribution of health facilities, and geographical characteristics. A program effectiveness comparison was easy because the Lahj governorate was SMHFPVP-supported while the Abyan governorate was not. The study selected districts and health facilities (HFs) in the Lahj governorate that provided reproductive health services (RHSs) with support from the SMHFPVP; in contrast, in the Abyan governorate, HFs were included that offered RHSs without support from the SMHFPVP. The study targeted mothers in both governorates who attended the targeted health facilities from January to December 2019; this was the period during which the SMHFPVP was fully implemented in the selected HFs in the Lahj governorate. The period of data collection was from July to December 2020.

### 2.2. Population Frame and Sample Size

The population frame was prepared by the researcher and the head of the Statistical Unit at both Governorate Health Offices (GHOs), with the help of an expert from the Central Statistical Organization (CSO) in the Lahj and Abyan governorates and the SMHFPVP coordinator in Lahj. In the Abyan governorate, the study targeted only HFs providing RHSs within districts with the same characteristics and the same number as the districts in the Lahj governorate, which did not receive any support from donors during 2019. This was conducted with full coordination with the Abyan GHO and Reproductive Health (RH) Department. In both governorates, the study included all governorate hospitals (GHs), district hospitals (DHs), maternal care health centers (MCHCs), health centers (HCs), and private hospitals (PHs) from the selected districts that fulfilled the inclusion criteria. Regarding health units (HUs), only 50% from each district fulfilled the inclusion criteria, from which sample units were selected using a simple random or systematic sampling technique.

Healthcare Facility (HF) Criteria:
Lahj Governorate:
Included: Public/private HFs providing RHSs (2018–2019), fully supported by the SMHFPVP in 2019, and not supported by other donors.
Excluded: HFs not providing RHSs, not supported by the SMHFPVP in 2019, or supported by other donors.
Abyan Governorate:
Included: Public/private HFs providing RHSs (2018–2019) and not supported by any donors.
Excluded: HFs not providing RHSs or supported by any donors.
Mother Selection Criteria (Reproductive Age 15–49)
Both Governorates: Mothers attending eligible HFs in 2019 until the sample size was reached.
Lahj Governorate:
Mothers who:
  - Had a child ≥2 years old prior to the study.
  - Received RHSs from SMHFPVP-supported HFs only.
  - Attended SMHFPVP-supported HFs for RHSs in 2019.
  - Lived in the same household (≥1 year) within the catchment area.
Abyan Governorate:
Mothers who:
  - Had a child ≥2 years old prior to the study.
  - Received RHSs from HFs with no donor support.
  - Lived in the same household (≥1 year) within the catchment area.

The sample preparation frame was based on RH reports from both governorates in 2019, illustrating the number and percentage of utilization of RHS packages among women who sought specific RH services in the targeted health facilities in both governorates, including ANC, facility-based delivery (FBD) (normal or cesarean section delivery), PNC, family planning (FP) services, and, finally, treatment of pregnancy-related complications (PRCs), for each targeted HF. This frame was used to calculate the prevalence of different RHSs per HF and district. By using the formula below, the required number of mothers was calculated for each HF and district. Subsequently, the required number of mothers comprising the required sample size in the selected HFs and districts was determined proportionately to the prevalence of RHSs and mothers who attended the HFs. The sample units (HFs) were selected using a simple random sampling technique.

All HFs in the targeted districts from both governorates that provided RHSs were listed. The number and the percentage of mothers who attended each of the targeted HFs from the analyzed districts during 2019 were determined in order to obtain the prevalence of utilization of each type of RHS in each HF during 2019. Probability proportionate to sample size was used to select the required number of mothers who fulfilled the inclusion criteria to yield and determine the needed number of mothers per HF in each district. In all the selected HFs, eligible mothers were recruited to participate in a face-to-face interview.

The sample size of married women (mothers), aged 15–49 years, who attended HFs in the targeted districts and fulfilled the inclusion and exclusion criteria was calculated using the following formula:

n=k2pqd2

where n = the sample size of married women (mothers) aged 15–49 years old selected for the study; k = the standard of 1.96 at 95% certainty; p = the prevalence of ANC, delivery by skilled birth personnel (SBPs) (FBD and home delivery (HD), PNC, treatment of pregnancy-related complications (PRCs), and FP in the targeted districts; q = 1 – p; and d = the precision or error allowable, which, in our study, is 0.05.

The calculated sample size in the Lahj governorate (based on prevalence assumptions), from the targeted districts, was 369 mothers, and 10% was added to this sample size to compensate for possible non-response, reaching a total sample size of 406 mothers. From the Abyan governorate targeted districts, 374 mothers, as well as 10% additional participants, were recruited to compensate for possible non-response, with a total sample size of 411 mothers. The required number of mothers from each district was considered using the probability proportionate to the number of mothers who received ANC in each target; they were interviewed directly, face-to-face, at the HFs in those districts.

### 2.3. Study Instruments/Data Collection Techniques (Questionnaire)

Paper-based, structured, pre-designed, and pre-tested questionnaires were employed in face-to-face interviews with the targeted mothers. Before the study, these questionnaires were piloted for consistency and validity. The questionnaires were designed utilizing numerous sources of standard RH information and based on national child and family health surveys, while satisfaction measures were taken from past studies. The questionnaires for the mothers who attended HFs consisted of the following sections: [the distribution of the selected health facilities; the distribution of the opinions of the mothers interviewed toward HF characteristics and the socioeconomic and sociodemographic characteristics of the mothers; reproductive health and family planning service utilization by the mothers interviewed; mothers’ satisfaction items; an SMHFPVP-related section (only for targeted mothers from the Lahj governorate); and utilization of reproductive health and family planning services (RHFPSs) by mothers (ANC, delivery, PNC, and FP)].

To understand mothers’ RH behaviors and decision-making concerning the aforementioned topics, well-structured interviews were conducted in both governorates. We employed standardized satisfaction-evaluation measures to assess mothers’ satisfaction, including Bruce’s RHS quality points, in the questionnaire [11].

These were as follows: (1) the accessibility and environmental characteristics of the HFs; (2) the continuity and sustainability of healthcare in the HFs; (3) the communication skills of staff and interpersonal relationships; (4) the provision of health education in the HFs; (5) the quality of healthcare services provided by the HFs. A total of 29 questions were prepared for all five aspects, and these were tested for their validity and reliability. The perceptions of the respondents were measured and rated on a three-point Likert Scale consisting of (1) “Low Satisfaction”, (2) “Moderate Satisfaction”, and (3) “High Satisfaction”. Overall satisfaction was defined as the average score for the five measured aspects of satisfaction. The mean level of satisfaction was calculated by averaging the respondents’ ratings for the satisfaction parameters. If a mother’s response scored (2.34) or above, the client was considered to have a high level of satisfaction, whereas if she scored (1.66) or less, she was considered to have a low level of satisfaction, and if she scored (1.67–2.33), the client was considered to have an undecided level of satisfaction (Moderate). The questionnaires were reviewed by RH experts at the Faculty of Medicine and RH experts at the Ministry of Health in both governorates in order to evaluate the relevance (or face validity) and completeness (or content validity) of the questionnaire. Participants providing and receiving RH and FP services were asked to describe their perceptions and reasons for satisfaction with the quality of the services. After piloting for consistency and validity, the questionnaires were used in the study. To ensure that the mothers could understand the questions, pilot research was conducted with 20 responses from each group. These responses were excluded from the study, and certain questions were rephrased. To assess questionnaire reliability, Cronbach’s α was determined.

### 2.4. Data Processing and Analysis

After the researcher checked and coded the questionnaires, Statistical Package for Social Sciences (SPSS) version 21 was utilized to examine quantitative data. Normality was evaluated using histograms and Kolmogorov–Smirnov tests (*p* > 0.05). In bivariate analysis, a *t*-test was employed for normally distributed data and a Whitney U-test for non-normal data. Comparisons were made using chi-square analysis for categorical variables. Fisher’s exact test and Yates’s adjustment for small cell frequency were significant tests. Quantitative variables were expressed using arithmetic means ± standard deviations (SDs) for normally distributed data and median & interquartile range (IQR) for not normally distributed variables. All the statistical analyses considered *p*-values < 0.05 to be significant.

## 3. Results

### 3.1. The Distribution of the Selected Health Facilities and the Mothers Interviewed

Around half the selected health facilities were health units in both governorates, followed by health centers, which represented around one-third (29.5%) in the Lahj governorate and 17.3% in the Abyan governorate, while the remaining health facilities were nearly equally distributed. Around half (44.8%) of the mothers interviewed attended health centers in the Lahj governorate, compared with 19.5% in the Abyan governorate, and around one-third (30.4%) of the mothers attended district hospitals in the Abyan governorate, compared with 14.8% in the Lahj governorate. On the other hand, mothers’ attendance at the other types of health facilities was nearly equal in both governorates.

### 3.2. The Sociodemographic Characteristics of the Mothers Interviewed

Table 2 illustrates the sociodemographic characteristics of the mothers interviewed in the Lahj and Abyan governorates.

Regarding the sociodemographic characteristics of the mothers interviewed in both governorates, we found no statistically significant differences, including in the mother’s age and education level, husband’s education level, and monthly family income; this indicates that these variables had no effect on access to and utilization of reproductive health services (RHSs) in either governorate.

### 3.3. The Opinions of the Mothers Interviewed Regarding the Characteristics of the Health Facilities

Table 3 demonstrates the distribution of the opinions of the mothers interviewed who attended HFs seeking RHSs. While the reported accessibility and the time to reach health facilities did not differ significantly between the Lahj and Abyan governorates (*p* = 0.189 and *p* = 0.471, respectively), transportation methods varied notably. Renting a car—funded by the SMHFPVP—was more common in Lahj (75.6%) than Abyan (58.9%), whereas walking was more frequent in Abyan (32.8%) than Lahj (19.0%) (both *p* < 0.001). Lahj also had significantly higher rates of routine 8 h workdays (66.5% vs. 52.3%) and more suitable working conditions (95.6% vs. 85.6%) (both *p* < 0.001). No significant differences were found in staff availability or waiting time suitability.

### 3.4. Family Planning (FP) Services

Table 4 illustrates the characteristics of the family planning (FP) methods utilized by the mothers interviewed in the Lahj and Abyan governorates. Mothers’ knowledge of FP methods and their acceptance of FP utilization were high in both governorates; however, they were significantly higher in the Lahj governorate compared to in the Abyan governorate. There was a non-statistically significant difference regarding previous FP utilization and the type of FP used. Modern FP methods (oral contraceptive pills, implants, injectables, contraceptive patches, vaginal rings, and intrauterine devices (IUDs)), and public health facilities as sources of FP utilization were both significantly higher in the Lahj governorate compared to in the Abyan governorate.

### 3.5. The Gestational History of the Mothers Interviewed in Both Governorates

Regarding the gestational history of the mothers interviewed in the voucher and non-voucher governorates, all the studied variables showed no statistically significant differences between both groups, as shown in Table 5 below.

### 3.6. Antenatal Care Service (ANC) Utilization in Both Governorates

Table 6 describes mothers’ utilization of ANC services as part of RHSs. In the table, all the variables showed that there were highly significant differences between the Lahj governorate and the Abyan governorate regarding the utilization of ANC services.

### 3.7. The Last-Delivery Characteristics of the Mothers Interviewed in Both Governorates

Table 7 demonstrates the last-delivery characteristics among the mothers interviewed in both governorates. Cesarean section (CS) delivery was significantly more frequently undertaken in the Lahj governorate compared to in the Abyan governorate (22.2% vs. 11.9%, *p* < 0.001); health facility delivery (public/private) was significantly higher in the Lahj governorate compared to in the Abyan governorate (88.2% vs. 62.5%, *p* < 0.001); delivery with trained healthcare providers (physician/ midwife/nurses) was significantly more frequent in the Lahj governorate compared to in the Abyan governorate (99.0% vs. 80.3%, *p* < 0.001); and free-of-charge delivery services were significantly more frequent in the Lahj governorate compared to in the Abyan governorate (95.8% vs. 32.8%, *p* < 0.001).

### 3.8. Postnatal Care (PNC) Service Utilization in Both Governorates

The utilization of PNC services was significantly higher in the Lahj governorate compared to in the Abyan governorate (73.9% vs. 25.1%, *p* < 0.001),

Table 8 demonstrates the postnatal care (PNC) utilization among the mothers interviewed in both governorates. Health facility PNC (public /private) was significantly more commonly used in the Lahj governorate compared to in the Abyan governorate (72.3% vs. 51.5%, *p* < 0.001); PNC with physicians was significantly more common in the Lahj governorate compared to in the Abyan governorate (58.7% vs. 22.3%, *p* < 0.001); the number of PNC visits was significantly higher in the Lahj governorate compared to in the Abyan governorate; and free-of-charge PNC services were significantly more prevalent in the Lahj governorate compared to in the Abyan governorate (98.3% vs. 38.8%, *p* < 0.001).

### 3.9. Neonatal Care (NNC) Service Utilization in Both Governorates

Table 9 demonstrates the neonatal care (NNC) utilization among the mothers interviewed in both governorates. NNC utilization was significantly higher in the Lahj governorate compared to in the Abyan governorate (63.5% vs. 24.6%, *p* < 0.001); health facility NNC (public /private) was significantly more common in the Lahj governorate compared to in the Abyan governorate (76.0% vs. 45.5%, *p* < 0.001); NNC with physicians was substantially more utilized in the Lahj governorate compared to in the Abyan governorate (50.4% vs. 28.7%, *p* < 0.001); the number of NNC visits was significantly higher in the Lahj governorate compared to in the Abyan governorate; and free-of-charge NNC services were significantly more prevalent in the Lahj governorate compared to in the Abyan governorate (97.7% vs. 40.6%, *p* < 0.001).

### 3.10. Mothers’ Satisfaction with Various Characteristics of Healthcare Facilities

Table 10 and Table 11 illustrate the mothers’ satisfaction with the characteristics of the health facility where the RHSs were accessed. As shown in the two tables, in all the items in the sections, the differences in means and percentages between both governorates are statistically significant (*p* < 0.001).

Almost all interviewed mothers in Lahj reported receiving and using ANC services provided by the SMHFPVP (98.5%); however, this utilization rate dwindled to 73.8% for PNC services and 63.5% for NNC services. Regarding family planning services, 76.6% of the mothers received an FP voucher and used it, while 9.6% received one through the SMHFPVP but did not use it, and the remaining 13.8% did not receive one and did not care about FP vouchers in general (Table 12).

**Figure 1:**
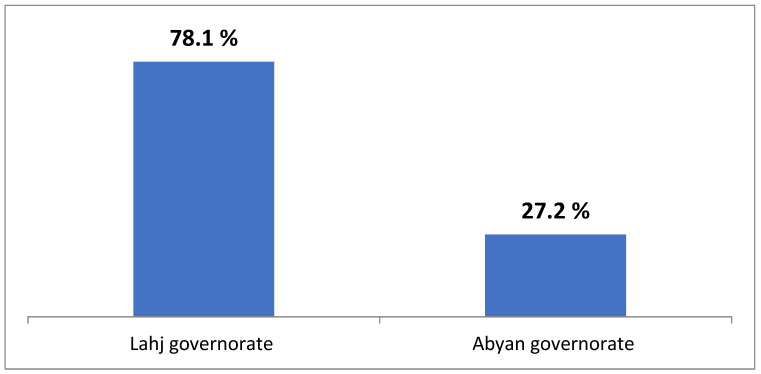
The percentage of the interviewed mothers who were satisfied with the health facility they used in the two governorates.

**Figure 2:**
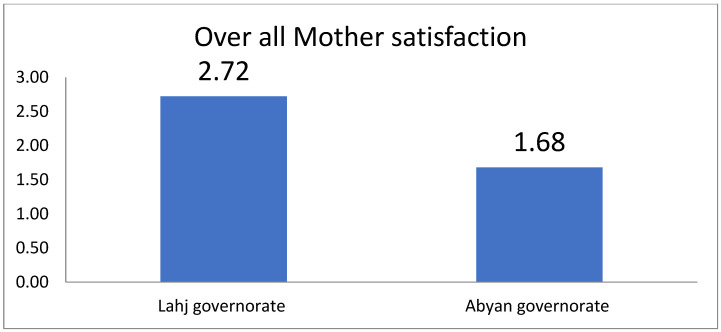
The difference in the overall satisfaction scores of the interviewed mothers between the two governorates.

Figure 1 and Figure 2 illustrate the overall satisfaction of mothers with the health facility where they received their RHSs. As shown in the two figures, the difference in the overall mean satisfaction score between both governorates was statistically significant, with *p*-values < 0.001.

## 4. Discussion

Access to and the cost of private primary care services are especially concerning in Yemen due to the undersupply of public primary care services and the financial obstacles caused by out-of-pocket payments [12]. There are three main benefits to the maternal health voucher program: products or services related to maternal and neonatal health, transportation, and accommodation.

In our study, the Lahj governorate demonstrated significantly superior RHS utilization and satisfaction compared to Abyan, despite Abyan having slightly better socioeconomic qualities, such as a higher wealth quintile and a higher rate of literacy. When analyzing the demographic data of our participants, we found that both governorates were statistically comparable, with no significant differences between the mothers interviewed in both regions regarding age (*p* = 0.508), education (*p* = 0.813), the husband’s education (*p* = 0.079), or monthly family income (*p* = 0.094), indicating that the disparities stemmed from health system factors, namely the SMHFPVP, rather than population characteristics. Additionally, mothers in Lahj had substantially higher rates of antenatal care (98.5% vs. 77.6%), facility-based deliveries (88.2% vs. 62.5%), postnatal care (73.9% vs. 25.1%), and neonatal care (63.5% vs. 24.6%), with most services being free-of-charge in Lahj, with the help of the SMHFPVP (72.3% free ANC, 95.8% free deliveries, 98.3% free PNC, and 97.7% NNC services, vs. 34.5%, 32.8%, 38.8%, and 40.6% in Abyan, respectively; *p* < 0.001).

Of the studied variables, statistically meaningful differences were noted in terms of methods of transportation, working hours, and workplace conditions. Our analysis revealed that 75.6% of mothers in Lahj utilized rental cars to commute to the healthcare facility (paid for by the SMHFPVP), as opposed to 58.9% of mothers in Abyan (who had no financial support by any program). Moreover, a significantly larger number of mothers in Abyan resorted to walking to the healthcare facility compared to in Lahj (32.8% vs. 19%; *p* < 0.001). Evidently, having a reliable means of transportation is critical for individuals to be able to access health centers and effectively use reproductive health services. Considering this, the availability of transportation becomes pivotal. The significantly higher rate of rental car use among Lahj mothers could be explained by the fact that the SMHFPVP in the Lahj governorate finances the transportation costs of its targeted mothers so that they may commute to and from medical appointments. Mothers in the Lahj governorate were probably able to overcome mobility obstacles and access reproductive health services more readily than mothers in the Abyan governorate thanks to this financial assistance, which made rental cars more accessible and inexpensive. However, women in the Lahj governorate (supported by the SMHFPVP) and the Abyan governorate (unsupported) did not differ significantly in their overall reports of accessibility of health facilities, with 72.2% of women in Lahj reporting easy accessibility compared to 70.3% in Abyan (*p*= 0.189).

Studies that have found women to be financially burdened by travel expenses indicate that transportation is a crucial component of maternal health voucher programs [13]. In 2018, 8.8 million Yemenis (30.6%) lived more than 30 min from the nearest partially or fully functional public primary healthcare facility, and 12.1 million (42.4%) lived more than 1 h away [14], which makes the results of our current study consistent with many previous studies in the same context. According to Tappis et al., financial access often hinders care-seeking more than physical access [15]. As expected, our study found that transportation is a major factor in the decision to use a health facility for ANC and delivery services. Mothers who used rental cars had an increased probability of using health facility delivery services as seen in Lahj governorate because the program covered the cost of rental cars compared with mother in Abyan where the program was not implemented.

In several Yemeni provinces and districts, the distance between health facilities and population housing, poor road infrastructure, and lack of transportation prevent people from using needed RH services [14]. A recent qualitative study examined a situation analysis for the provision of integrated comprehensive sexual and reproductive health services to Yemeni displaced people, stating that “Geographic distance and challenging roads with the scarcity of transportation and its high cost ranging from 20,000 to 30,000 YRs in this financial difficulty has prohibited women from accessing health care services.” [16].

A comprehensive assessment of eight conflict-affected countries, including Yemen, examined maternal health service consumption from both perspectives: the demand (community) angle included mobility, female education, autonomy, health awareness, and ability to pay, whereas the supply (health services) angle included service availability and quality, community health workers, and health facility charges and informal payment [17].

The survey found considerable differences in the availability of health facilities between the governorates. A higher number of health facilities in the Abyan governorate are available 24/7 than in Lahj (47.7% vs. 33.5%, *p* < 0.001). Despite this, targeted women in the Abyan governorate, which lacked program assistance, used RHSs less than in the Lahj governorate, which was supported by the SMHFPVP. This shows that variables other than health facility working hours affect mothers’ access to RHSs. Conversely, a Nigerian study revealed that inconvenient health facility opening hours prevented teenagers from using reproductive healthcare [18]. A Pakistani study found that health facilities’ operating hours prevented them from providing 24/7 necessary services, jeopardizing countless lives [19].

Onono et al., in their Kenyan study, found that even though women were aware of the benefits of skilled birth attendance, other systemic factors, like opening hours or healthcare workers’ attitudes, still deterred them from delivering in health facilities [20]. Facility operating hours and the restricted number of public facilities impeded access to healthcare [21].

Regarding reproductive health service characteristics and utilization, Yemen has one of the highest maternal death rates in the Arab world, 385 per 100,000 live births [8]; without access to reproductive health services, women have an increased risk of life-threatening complications [22].

A maternal and child health voucher scheme to subsidize four services—antenatal care (ANC), delivery, postnatal care (PNC), and neonatal care (NNC)—for pregnant women would help them to overcome financial barriers and would raise awareness of ANC and skilled birth attendant (SBA) delivery, which can reduce maternal and neonatal mortality. Lahj governorate mothers used ANC, delivery services by SBAs (at HFs and at home), PNC, and NNC services more than those in the Abyan governorate, and virtually all women received ANC. In Lahj, 82.5% of the interviewed mothers received ANC by a doctor, compared to 39.2% of the women in Abyan. ANC visits were likewise higher in Lahj than in the Abyan governorate. The majority (88.2%) of interviewed mothers in the Lahj governorate gave birth in a hospital attended by a trained healthcare provider, compared to 62.5% of the mothers in Abyan.

Yemen exhibits the lowest antenatal care coverage in the region, presenting numerous problems for women when it comes to institutional birth, resulting in most mothers delivering at home. The latest National Health and Demographic Survey (NHDS) for Yemen indicates that 34% of Yemeni women in rural regions receive assistance from a skilled birth attendant during childbirth, in contrast to 73% in urban areas, while only 23% of women in rural areas deliver at a health facility [23]. The factors influencing demand in Yemen for maternal health service (MHS) utilization are found to be affected by conflict, as poverty and the inability to pay, which have been worsened by the recent political situation in Yemen, cause women to neglect their health, assuming that if there are no obvious complications, it is not necessary to utilize maternal health services [17]. Previous research in Uganda, Kenya, and Pakistan has shown that coupons improve maternal care utilization [24,25,26].

In contrast, a quasi-experimental study in Kenya examined how a maternal health voucher program affected service utilization before and after free maternity services were introduced. The difference between their study and others may be due to free maternity services in non-voucher counties, which improved access to facility-based deliveries so significantly that outcomes in these areas nearly matched those in voucher-supported regions [27]. Voucher program coverage was associated with a two-fold increase in delivery at a health facility (OR: 2.1; 95% CI: 1.5–3.1) [28]. Cambodia’s voucher program improves institutional delivery. It has been linked to a 10% rise in institutional delivery and a 15.6% increase in public healthcare facility childbirth among the poorest 40% of households [29]; similar results have been found in studies in Kenya [30], Bangladesh, and Pakistan [25].

In Yemen, knowledge of family planning services is universally high, exceeding 98% across all demographics, including age, education, wealth, residency, and governorate. Women in rural areas report a lower level of knowledge (97.6%) compared to nearly all women in urban areas (99.8%) [23].

The findings of the current study indicate that in Lahj, knowledge (98.5% vs. 92.9%; *p* < 0.001), approval (95.8% vs. 87.4%; *p* < 0.001), and modern-method usage (92.3% vs. 67.8%; *p* < 0.001) were significantly higher compared to in Abyan, primarily sourced from public facilities (71.7% vs. 50.6%; *p* < 0.001). Modern methods of contraception (oral contraceptive pills, implants, injectables, contraceptive patches, vaginal rings, IUDs), were used at significantly greater rates in Lahj compared to in the Abyan governorate, whereas traditional family planning methods were more commonly used in Abyan (32.2% vs. 7.7%; *p* < 0.001), corroborating previous studies in Pakistan [23,24,25,26,27,28,29,30,31,32], also in other study done by Ali et al. reported a 26% increase in net percentage points for modern contraceptives in intervention areas and an 18% increase in points for IUD use was also noted [19].

In terms of uses the voucher for FP services—only for mothers in Lahj governorate were the program implemented—only 76.6% of mothers in Lahj received and used vouchers for FP services, while 9.6% received a voucher for FP, but did not use it. On the other hand, 13.8% of mothers did not care for FP vouchers, so they did not receive a voucher, and around a quarter of the mothers did not use FP services (23.4%), either because they did not receive a voucher, or they received a voucher and did not use it. Despite calls from the Ministry of Health to prioritize the distribution of family planning vouchers for free, this remains an underutilized service [33]. Targeting disadvantaged women with a locally contextualized (provincial or territorial) poverty assessment tool can improve voucher eligibility criteria for future voucher schemes.

Regarding mothers’ satisfaction, program monitoring and evaluation usually focus on service and voucher use. Some programs track quality indicators including patient satisfaction and provider knowledge. To measure mother satisfaction, we used established satisfaction-measuring instruments, including Bruce’s major criteria for RHS quality, in the questionnaire [11]. These were (1) the accessibility and environmental characteristics of the HFs, (2) the continuity and sustainability of healthcare in the HFs, (3) the communication skills of the staff and interpersonal relationships, (4) the provision of health education at the HFs, and (5) the quality of healthcare services at the HFs.

The current study found significantly higher satisfaction scores in the Lahj governorate—where vouchers were supported—compared to in the Abyan governorate—which did not implement the voucher program—across all satisfaction domains, as well as in the total satisfaction scores. The war in Yemen recently led to the months-long closure of many government facilities, including the governorate primary hospital, which explains these results. During wartime, nearby private midwifery clinics and hospitals have provided these reproductive health services to women.

The Safe Motherhood Voucher includes free family planning in its postnatal care package. User satisfaction has been investigated in many voucher program reviews. The Uganda Reproductive Health Vouchers Project (RHVP) pleased 94% of voucher users and 76% of non-voucher users. Coupon facilities also performed more comprehensive prenatal and postnatal clinical exams than non-voucher facilities [34]. In Uganda, 99% of family planning voucher recipients assessed the program as satisfactory or very good [35]. In Madagascar, 95% of voucher recipients were satisfied with the overall program [36]. According to Abuya et al., 99% of individuals who redeemed vouchers rated the services as good or very good [37].

Conversely, Kundu et al., in their study, reported that non-voucher users had higher levels of satisfaction than voucher users: 93.9% of non-voucher clients reported being satisfied or very satisfied with their service, vs. 88.4% of voucher users (*p* = 0.032) [38].

Also, the patient satisfaction scores in our study are consistent with many previous studies, which have shown that vouchers increase patient satisfaction as the user is at liberty to choose a provider [39]. When the voucher encompasses the total expense of services, or when the costs are the same across all providers, the holder typically makes a selection based on their impression of which provider delivers the most convenient, most comfortable, and highest-quality service [24]. This choice raises satisfaction with the service, and providers will consequently tend to raise the quality of their services to attract more voucher users [40].

The impact of vouchers on user satisfaction has been shown to be largely positive in most previous studies [24]. A thorough review identified benefits such as quality improvement and patient satisfaction. Therefore, it seems pertinent to evaluate the efficacy of voucher programs in relation to other alternative health financing methods [41].

## 5. Limitations

Our study faced some limitations, such as the ongoing conflict and instability, which limit access to some areas, especially in remote or conflict-prone districts (frontline health facilities). In addition, the large numbers of internally displaced persons (IDPs), including mothers, may skew population characteristics, as displacement affects access to health services and might make it hard to track consistent health behaviors or outcomes, especially in the Abyan governorate. Furthermore, variation in health facility quality between Lahj and Abyan may have influenced health outcomes independently of the voucher program. Lastly, some of the selected health facilities were non-functional due to the conflict, so they were replaced by other HFs.

## 6. Conclusions

The primary impacts of the voucher program in Yemen on healthcare providers and HFs included ensuring a consistent flow of funds to facilities amidst unreliable or discontinued government financing; maintaining service provision, even during the most critical phases of the crisis; enhancing local adaptability and capacity to manage drug and supply shortages; and facilitating quality-assurance support.

The vouchers enabled impoverished women and their families, frequently residing in isolated rural regions, to secure institutional deliveries without the burden of unforeseen and exorbitant expenses, such as travel with an escort, while enhancing provider selection by permitting women to choose a suitable healthcare provider nearer to their residence.

Although voucher programs provide benefits, they are not a robust health system strategy, and they fail to address the long-standing and deep-rooted health system challenges faced by fragile states. Nevertheless, fragility and instability frequently result in urgent health needs, particularly for women and adolescents, which must be addressed promptly and efficiently. We advise program implementers in precarious circumstances to utilize vouchers to sustain the provision of safe motherhood services to women in need.

## Figures and Tables

**Table 1:** Socio-demographic and health characteristics of mothers of reproductive age (15–49) in the Lahj and Abyan governorates.

Characteristic	Lahj	Abyan
Total population size	722,694	433,819
Percentage of population distribution in rural area	60–70%	50–60%
Wealth quintiles:		
Lowest:	19.8	6.4
Highest:	8.5	13.6
Percentages of female educational attainment:		
No education:	42.0	40.0
Fundamental:	43.3	50.4
Higher:	4.8	4.9
Percentages of male educational attainment:		
No education:	18.3	14.4
Fundamental:	53.7	56.1
Higher:	9.7	7.3
Percentage of literacy among women aged 15–49	54.5	62.7
Percentage of women aged 15–49 of employment status	16.0	17.5
Percentage of women exposed to mass media (newspapers, television, and radio)	8.0	11.4
Percentage of ever-married women	2.6	2.1
Percentage of never-married women	2.9	2.3
Total fertility rate	4.5	4.0
Median age at first birth	23.4	24.3
Mean ideal number of children	4.4	4.6
Wanted fertility rates	3.3	3.0
Percentage of mothers having heard of any method of FP	100	99.0
Percentage of mothers currently using any modern FP	30.4	29.5
Percentage of mothers receiving antenatal care from a skilled provider	67.0	70.5
Percentage of mothers having delivered in a health facility	41.0	57.5
Prevalence of anemia among females of reproductive age (15–49)	90.3	92.4

Source: Yemen National Health and Demographic Survey—2013.

**Table 2:** The sociodemographic characteristics of the mothers interviewed.

Characteristics	Lahj (N = 406)		Abyan (N = 411)		X^2^	*p*-Value
	No.	%	No.	%		
Age of mother:						
≤18 years	17	4.2	21	2.1	1.355	0.508
19–35 years	347	85.5	339	82.5		
<35 years	42	10.3	51	12.4		
Median (IQR)	27 (23–32)		28 (23–33)			0.185 ^#^
Mother’s education level:						
Illiterate	78	19.2	74	18.0	0.413	0.813
Below university	258	63.6	270	65.7		
University degree and above	70	17.2	67	16.3		
Husband’s education level:						
Illiterate	38	9.4	25	6.1	5.077	0.079
Below	278	68.4	274	66.6		
University degree and above	90	22.2	112	27.3		
Monthly family income (YER *):						
<40,000 RY	83	20.4	100	24.3	4.734	0.094
40,000–80,000 RY	280	69.0	283	68.9		
>80,000 RY	43	10.6	28	6.8		
Median (IQR)	60,000 (50,000-70,000)		60,000 (45,000-70,000)			0.070 ^#^

* Yameni Rial; ^#^ Statistical analysis by Mann–Whitney U test.

**Table 3:** The distribution of the opinions toward health facility characteristics of the mothers interviewed who attended HFs seeking RHSs.

Characteristics	Lahj (N = 406)		Abyan (N = 411)		X^2^	*p*-Value
	No.	%	No.	%		
Physical accessibility						
Easily accessible	293	72.2	289	70.3	3.336	0.189
In between	42	10.3	59	14.4		
Difficult to access	71	17.5	63	15.3		
Method of transportation						
Walking	77	19.0	135	32.8	26.106	<0.001 *
Car rental	307	75.6	242	58.9		
Private car	22	5.4	34	8.3		
Time taken to reach HF:						
≤30 min	287	70.7	281	68.4	0.519	0.471
>30 min	119	29.3	130	31.6		
Median (IQR)	30 (30–40)		30 (25–40)			0.953 ^#^
Working hours:						
Routine working hours (8 h/day)	270	66.5	215	52.3	17.051	<0.001 *
24 h/day	136	33.5	196	47.7		
Availability of health staff:						
Always available	353	86.9	361	87.8	0.146	0.702
Not always available	53	13.1	50	12.2		
Suitability of workplace conditions:						
Not suitable	5	1.2	8	1.9	24.976	<0.001 *
Somewhat suitable	13	3.2	51	12.4		
Suitable	388	95.6	352	85.6		
Suitability of waiting time at health facility:						
Not suitable	19	4.7	15	3.6	2.253	0.324
Somewhat suitable	34	8.4	46	11.2		
Suitable	353	86.9	350	85.2		

Chi-square (Χ^2^) tests. * *p*-value < 0.001 is statistically significant. ^#^ Statistical analysis by Mann–Whitney U test.

**Table 4:** Mothers’ attitudes and practices toward family planning in the Lahj and Abyan governorates.

Characteristics	Lahj		Abyan		X^2^	*p*-Value
	No.	%	No.	%		
Mothers’ knowledge of FP	(n = 406)		(n = 411)			
Had heard of any FP methods	400	98.5	382	92.9	15.499	<0.001 *
Had not heard of any FP methods	6	1.5	29	7.1		
Mothers’ previous use of FP	(n = 400)		(n = 382)			
Yes	264	66.0	250	65.4	2.131	0.144
No	136	34.0	132	34.6		
Type of FP Previously Used	(n = 264)		(n = 250)			
Modern FP **	172	65.2	167	66.8	4.104	0.128
Traditional FP	92	34.8	83	33.2		
Mothers’ approval of FP use	(n = 400)		(n = 382)			
Yes	383	95.8	334	87.4	35.601	<0.001 *
No	12	3.0	22	5.8		
Not her decision	5	1.2	26	6.8		
Current use of FP method	(n = 383)		(n = 334)			
Yes	337	88.0	227	68.0	14.488	<0.001 *
No	46	12.0	107	32.0		
Type of FP currently used	(n = 337)		(n = 227)			
Modern FP	311	92.3	154	67.8	31.022	<0.001 *
Traditional FP	26	7.7	73	32.2		
Source of FP methods	(n = 311)		(n = 154)			
Public health facility	223	71.7	78	50.6	56.462	<0.001 *
Private health facility	88	28.3	76	49.4		

Chi-square (Χ^2^) tests. * *p*-value < 0.001 is statistically significant. ** Modern family planning methods include oral contraceptive pills, implants, injectables, contraceptive patches, vaginal rings, and intrauterine devices (IUDs).

**Table 5:** Characteristics of past gestational history of mothers interviewed, in Lahj governorate and Abyan governorate.

Characteristics	Lahj (n = 406)		Abyan (n = 411)		X^2^	*p*-Value
	No.	%	No.	%		
Age of mother at marriage						
≤18 years	174	42.9	176	42.8	6.029	0.059
19 to 25 years	185	45.5	207	50.4		
<25 years	47	11.6	28	6.8		
Median (IQR)	19 (17–23)		19 (17–21)			0.085 ^#^
Age of mother at first delivery						
≤18 years	107	26.4	110	26.8	0.018	0.991
19 to 35 years	298	73.4	300	73		
>35 years	1	0.2	1	0.2		
Median (IQR)	20 (18–24)		20 (18–23)			0.466 ^#^
Number of pregnancies mother experienced during reproductive age						
<4 pregnancies	302	74.4	301	73.2	0.169	0.919
4 to 7 pregnancies	88	21.7	94	22.9		
>7 pregnancies	16	3.9	16	3.9		
Median (IQR)	3 (2–5)		3 (2–5)			0.058 ^#^

Chi-square (Χ^2^) tests. ^#^ Statistical analysis by Mann–Whitney U test.

**Table 6:** Antenatal care (ANC) service utilization by the mothers interviewed in the Lahj and Abyan governorates.

Characteristics	Lahj		Abyan		X^2^	*p*-Value
	No.	%	No.	%		
Mother’s utilization of ANC services	(n = 406)		(n = 411)			
Yes	400	98.5	319	77.6	84.567	<0.001 *
No	6	1.5	92	22.4		
Location of ANC services	(n = 400)		(n = 319)			
At health facility	400	100	272	85.3	146.825	<0.001 *
At home	0	0.0	47	14.7		
Who provided last ANC services	(n = 400)		(n = 319)			
Physician	330	82.5	125	39.2	226.052	<0.001 *
Midwife/nurse	70	17.5	194	60.8		
Number of ANC services	(n = 400)		(n = 319)			
Four visits or more	279	69.8	143	44.8		<0.001 *
Three visits	57	14.3	24	7.5	171.63	
Two visits	49	12.3	96	30.1		
One visit	15	3.8	56	17.6		
Payment to receive ANC services	(n = 400)		(n = 319)			
With fees—cash	111	27.8	209	65.5	185.762	<0.001 *
Free of charge	289	72.3	110	34.5		
Vaccination with TT	(n = 406)		(n = 411)			
Not vaccinated	67	16.5	151	36.7	42.757	<0.001 *
Vaccinated	339	83.5	260	63.3		
Number of doses of TT	(n = 339)		(n = 260)			
Five doses	74	21.8	38	14.6	92.251	<0.001 *
Four doses	86	25.4	29	11.2		
Three doses	104	30.7	80	30.8		
Two doses	59	17.4	64	24.6		
One dose	16	4.7	49	18.8		

Chi-square (Χ^2^) tests. * *p*-value < 0.001 is statistically significant.

**Table 7:** The last-delivery circumstances of the mothers interviewed in the Lahj and Abyan governorates, 2020.

Characteristics	Lahj (N = 406)		Abyan (N = 411)		X^2^	*p*-Value
	No.	%	No.	%		
Nature of last delivery:						
Normal delivery	316	77.8	362	88.1	15.184	<0.001 *
Cesarean section delivery	90	22.2	49	11.9		
Place of last delivery:						
Health facility delivery (public /private)	358	88.2	257	62.5	72.183	<0.001 *
Home delivery	48	11.8	154	37.5		
Who provided delivery:						
Trained healthcare provider	402	99.0	330	80.3	76.807	<0.001 *
Traditional birth attendant (Daya)	4	1.0	81	19.7		
Payment to receive delivery and delivery-related complication treatment:						
With fees—cash	17	4.2	276	67.2	352.050	<0.001 *
Free of charge	389	95.8	135	32.8		

Chi-square (Χ^2^) tests. * *p*-value < 0.001 is statistically significant.

**Table 8:** Comparison of postnatal care (PNC) utilization by mothers interviewed in Lahj and Abyan governorates, 2020.

Characteristics	Lahj (N = 300)		Abyan (N = 103)		X^2^	*p*-Value
	No.	%	No.	%		
Utilization of PNC services						
Yes	300	73.9	103	25.1	194.837	<0.001 *
No	106	26.1	308	74.9		
Location of PNC services						
Health facility delivery	220	72.3	53	51.5	207.618	<0.001 *
Home delivery	80	26.7	50	48.5		
Who provided PNC services						
Physician	176	58.7	23	22.3	225.662	<0.001 *
Midwife/nurse	124	41.3	80	77.7		
Number of PNC visits						
Three visits or more	164	54.7	24	23.3	254.021	<0.001 *
Two visits	99	33.0	23	22.3		
One visit	37	12.3	56	54.4		
Payment to receive PNC services						
With fees—cash	5	1.7	63	61.2	342.118	<0.001 *
Free of charge	295	98.3	40	38.8		

Chi-square (Χ^2^) tests. * *p*-value <0.001 is statistically significant.

**Table 9:** Comparison of neonatal care (NNC) utilization by mothers interviewed in Lahj and Abyan governorates, 2020.

Characteristics	Lahj		Abyan		X^2^	*p*-Value
	No.	%	No.	%		
Utilization of NNC services	(n = 406)		(n = 411)			
Yes	258	63.5	101	24.6	126.714	<0.001 *
No	148	36.5	310	75.4		
Location of NNC services	(n = 258)		(n = 101)			
Health facility delivery	196	76.0	46	45.5	150.670	<0.001 *
Home delivery	62	24.0	55	54.5		
Who provided NNC services	(n = 258)		(n = 101)			
Physician	130	50.4	29	28.7	137.113	<0.001 *
Midwife/nurse	128	49.6	72	71.3		
Number of NNC visits	(n = 258)		(n = 101)			
Three visits or more	142	55.1	30	29.7	151.981	<0.001 *
Two visits	69	26.7	24	23.8		
One visit	47	18.2	47	46.5		
Payment to receive NNC services	(n = 258)		(n = 101)			
With fees—cash	6	2.3	60	59.4	253.441	<0.001 *
Free of charge	252	97.7	41	40.6		

* p-value < 0.001 is statistically significant.

**Table 10:** Mothers’ satisfaction with the health facilities in the two governorates studied.

Characteristics	Lahj (406)		Abyan (411)		*p*-Value
	No.	%	No.	%	
Mothers’ satisfaction with accessibility and environmental characteristics of health facility:					
1—Infrastructure conditions					
Highly Satisfied	321	79.0	114	27.7	<0.001 *
2—Availability of drugs and equipment					
Highly Satisfied	344	84.7	105	25.5	<0.001 *
3—Location (accessibility)					
Highly Satisfied	315	77.6	111	27.0	<0.001 *
4—Suitability of waiting area					
Highly Satisfied	328	80.8	117	28.4	<0.001 *
5—Suitability of waiting time					
Highly Satisfied	313	77.1	115	28.0	<0.001 *
6—Male/female waiting area separated					
Highly Satisfied	313	77.1	108	26.3	<0.001 *
7—Right to select medical staff					
Highly Satisfied	342	84.2	97	23.6	<0.001 *
8—Staff experience					
Highly Satisfied	315	77.6	88	21.4	<0.001 *
9—Proper staff response					
Highly Satisfied	317	78.1	111	27.0	<0.001 *
Overall response for first section					
Highly Satisfied	323	79.6	107	26.1	<0.001 *
Mothers’ satisfaction with continuity and sustainability of healthcare at health facility:					
1—Availability of needed services					
Highly Satisfied	277	68.2	99	24.0	<0.001 *
2—Availability of referral system					
Highly Satisfied	302	74.4	113	27.5	<0.001 *
3—Contacted and followed up at home					
Highly Satisfied	314	77.3	114	27.8	<0.001 *
4—Suitability of working hours					
Highly Satisfied	314	77.3	115	28.0	<0.001 *
5—Availability of experienced staff					
Highly Satisfied	299	73.7	109	26.5	<0.001 *
Overall response for second section					
Highly Satisfied	301	74.2	110	26.8	<0.001 *
Mothers’ satisfaction with communication skills of staff and their interpersonal relationships:					
1—Staff were respectful and polite					
Highly Satisfied	273	67.2	124	30.2	<0.001 *
2—Administrate and manager response to patient complaints					
Highly Satisfied	282	69.4	128	31.1	<0.001 *
3—Respect for patient privacy					
Highly Satisfied	317	78.1	115	28.0	<0.001 *
4—Staff response to patients’ questions about their case					
Highly Satisfied	319	78.6	106	25.8	<0.001 *
5—Confidence with staff					
Highly Satisfied	305	75.1	100	24.3	<0.001 *
Overall response for the third section					
Highly Satisfied	299	73.7	115	27.9	<0.001 *
Mothers’ satisfaction with provision of health education:					
1—Availability of health education					
Highly Satisfied	342	84.3	115	26.3	<0.001 *
2—Pharmacist’s role is performed properly					
Highly Satisfied	315	77.6	116	28.2	<0.001 *
3—Health staff promotes mothers to use RHSs					
Highly Satisfied	335	82.5	103	25.1	<0.001 *
4—Health education provided through experienced staff					
Highly Satisfied	344	84.7	100	24.3	<0.001 *
Overall response for fourth section					
Highly Satisfied	334	82.3	109	24.4	<0.001 *
Mothers’ satisfaction with quality of healthcare services:					
1—The health facility has a proper filing system					
Highly Satisfied	315	77.6	111	27.0	<0.001 *
2—The health facility easily handles medical documents					
Highly Satisfied	314	77.3	127	30.9	<0.001 *
3—Staff dedicate enough time to the case					
Highly Satisfied	349	86.0	127	30.9	<0.001 *
4—The necessary medication is provided easily and for free					
Highly Satisfied	351	86.5	118	28.7	<0.001 *
5—The HF is always clean, tidy, and well organized					
Highly Satisfied	310	76.4	115	28.0	<0.001 *
6—Satisfaction with the health services					
Highly Satisfied	340	83.7	110	26.8	<0.001 *
Overall response for fifth section					
Highly Satisfied	330	81.2	118	28.7	<0.001 *
Overall response for all sections					
Highly Satisfied	317	78.1	112	27.2	<0.001 *

* *p*-value < 0.001 is considered statistically significant.

**Table 11:** Differences in mothers’ satisfaction with the health facilities in the two governorates studied.

Characteristics	Lahj, (N = 406)	Abyan, (N = 411)	*p*-Value
Mothers’ satisfaction with accessibility and environmental characteristics of health facility:			
Infrastructure conditions	2.60 ± 0.788	1.67 ± 0.881	<0.001 *
Availability of drugs and equipment	2.74 ± 0.646	1.62 ± 0.865	<0.001 *
Location (accessibility)	2.60 ± 0.765	1.64 ± 0.879	<0.001 *
Suitability of waiting area	2.65 ± 0.741	1.66 ± 0.893	<0.001 *
Suitability of waiting time	2.60 ± 0.766	1.70 ± 0.879	<0.001 *
Male/female waiting area separated	2.61 ± 0.748	1.65 ± 0.868	<0.001 *
Right to select medical staff	2.74 ± 0.633	1.58 ± 0.847	<0.001 *
Staff experience	2.61 ± 0.762	1.47 ± 0.824	<0.001 *
Proper staff response	2.60 ± 0.775	1.68 ± 0.872	<0.001 *
Overall response for first section	2.64 ± 0.457	1.63 ± 0.406	<0.001 *
Mothers’ satisfaction with continuity and sustainability of healthcare at health facility:			
Availability of needed services	2.52 ± 0.762	1.59 ± 0.852	<0.001 *
Availability of referral system	2.57 ± 0.765	1.67 ± 0.879	<0.001 *
Contacted and followed up at home	2.61 ± 0.755	1.64 ± 0.888	<0.001 *
Suitability of working hours	2.63 ± 0.729	1.69 ± 0.881	<0.001 *
Availability of experienced staff	2.55 ± 0.790	1.64 ± 0.674	<0.001 *
Overall response for second section	2.57 ± 0.477	1.64 ± 0.504	<0.001 *
Mothers’ satisfaction with communication skills of staff and their interpersonal relationships:			
Staff were respectful and polite	2.43 ± 0.857	1.69 ± 0.906	<0.001 *
Administration and manager response to patient complaints	2.47 ± 0.833	1.78 ± 0.89	<0.001 *
Respect for patient privacy	2.66 ± 0.692	1.69 ± 0.880	<0.001 *
Staff response to patient’s questions about their case	2.64 ± 0.719	1.63 ± 0.866	<0.001 *
Confidence with staff	2.58 ± 0.765	1.62 ± 0.851	<0.001 *
Overall response of the third section	2.56 ± 0.488	1.68 ± 0.484	<0.001 *
Mothers’ satisfaction with provision of health education:			
Availability of health education	2.76 ± 0.598	1.61 ± 0.874	<0.001 *
Pharmacist’s role performed properly	2.61 ± 0.762	1.67 ± 0.888	<0.001 *
Health staff promote mothers to use RHSs	2.77 ± 0.546	1.6 ± 0.862	<0.001 *
Health education provided by experienced staff	2.76 ± 0.606	1.59 ± 0.854	<0.001 *
Overall response for fourth section	2.72 ± 0.404	1.62 ± 0.489	<0.001 *
Mothers’ satisfaction with quality of healthcare services:			
The health facility has a proper filing system	2.7 ± 0.598	1.66 ± 0.876	<0.001 *
Easy handling of medical documents in the health facility	2.68 ± 0.628	1.71 ± 0.909	<0.001 *
Staff dedicate enough time to the case	2.77 ± 0.590	1.73 ± 0.905	<0.001 *
The necessary medication is provided easily and for free	2.79 ± 0.566	1.68 ± 0.892	<0.001 *
The HF is always clean, tidy, and well organized	2.62 ± 0.727	1.67 ± 0.884	<0.001 *
Satisfaction with health services	2.74 ± 0.630	1.64 ± 0.875	<0.001 *
Overall response for fifth section	2.72 ± 0.346	1.68 ± 0.515	<0.001 *

* *p*-value < 0.05 is statistically significant. Statistical analysis by Mann–Whitney U test.

**Table 12:** Types of RHSs provided and covered by Safe Motherhood Family Planning Voucher Program (SMHFPV), in Lahj governorate only.

Items	Studied Mothers in Lahj Governorate	
	No.	%
ANC service utilization		
SMHFPVP voucher received and used	400	98.5
Did not use voucher for ANC	6	1.5
Nature and location of delivery services covered by voucher		
Normal delivery at a public HF	260	64.5
Normal delivery at a private HF	8	2
Normal delivery at home by an SBA	48	11.3
Cesarean section at a public health facility	78	19.2
Cesarean section at a private health facility	12	3
PNC service utilization covered by voucher		
PNC services at a public HF	202	49.8
PNC services at a private HF	18	4.4
PNC services provided at home by an SBA	80	19.7
Did not use voucher for PNC	106	26.1
NNC services		
NNC services at a public HF	173	45.1
NNC services at a private HF	23	5.8
NNC services provided at home by an SBA	62	13.4
Did not use voucher	148	36.5
FP services		
Accessed and used FP through SMHFPVP	311	76.6
Gained access to but did not use FP	39	9.6
Not received (did not care about FP vouchers)	56	13.8

## Data Availability

The datasets used and/or analyzed during the current study are available from the corresponding author on reasonable request.
